# The reported impact of public involvement in biobanks: A scoping review

**DOI:** 10.1111/hex.13067

**Published:** 2020-05-06

**Authors:** Lidia Luna Puerta, Will Kendall, Bethan Davies, Sophie Day, Helen Ward

**Affiliations:** ^1^ NIHR Imperial BRC Patient Experience Research Centre Imperial College London London UK; ^2^ Family Medicine and Primary Care Lee Kong Chian School of Medicine Nanyang Technological University Singapore Singapore Singapore; ^3^ Department of Sociology London School of Economics London UK

**Keywords:** biobank, impact, public involvement

## Abstract

**Background:**

Biobanks increasingly employ public involvement and engagement strategies, though few studies have explored their impact. This review aims to (a) investigate how the impact of public involvement in biobanks is reported and conceptualized by study authors; in order to (b) suggest how the research community might re‐conceptualize the impact of public involvement in biobanks.

**Methods:**

A systematic literature search of three electronic databases and the INVOLVE Evidence Library in January 2019. Studies commenting on the impact of public involvement in a biobank were included, and a narrative review was conducted.

**Results and discussion:**

Forty‐one studies covering thirty‐one biobanks were included, with varying degrees of public involvement. Impact was categorized according to *where* it was seen: ‘the biobank’, ‘people involved’ and ‘the wider research community’. Most studies reported involvement in a ‘functional’ way, in relation to improved rates of participation in the biobank. Broader forms of impact were reported but were vaguely defined and measured. This review highlights a lack of clarity of purpose and varied researcher conceptualizations of involvement. We pose three areas for further research and consideration by biobank researchers and public involvement practitioners.

**Conclusions:**

Functional approaches to public involvement in biobanking limit impact. This conceptualization of involvement emerges from an entrenched technical understanding that ignores its political nature, complicated by long‐standing disagreement about the values of public involvement. This study urges a re‐imagination of impact, re‐conceptualized as a two‐way learning process. More support will help researchers and members of the public to undergo such reflective exercises.

**What is already known on this topic**

- Involving the public in research helps improve research and maintain trust
- The methods and tasks of involving the public are varied
- There is limited evidence of the nature and scale of the impact of involvement in biobanks

**What this study adds**

- There is no consensus about the objectives of public involvement in biobanks, and this undermines the ability to measure impact
- The majority of biobank studies report public involvement in a ‘functionalist’ way, with the purpose of improving recruitment processes and participation rates. Accordingly, the impact centres around functional domains such as enhancement of consent forms, processes and recruitment
- There are significant epistemological and methodological challenges to capturing impact, suggesting that the impact of public involvement needs to be re‐imagined, both in biobanking and wider health and social care research

## 1 INTRODUCTION

### 1.1 Biobanks

Biobanks are large collections of samples linked to data which today combine genetic, medical and other personal data.^1^ They entail the collection and storage of tissue and/or blood samples, and often require additional personal data, such as genealogical and lifestyle information.^2^ Biobanks vary according to tissue type, purpose of use and ownership.^3^ They are operated by a variety of actors, ranging from hospitals and research institutes to pharmaceutical companies and patient organizations.

The practice of biobanking presents a series of ethical, legal and social implications (ELSI),^4^ including ‘the fairness of collecting [or failing to collect] donations from vulnerable populations, providing informed consent [open or re‐consented each time or one off] to donors, the logistics of data disclosure to participants, the right to ownership of intellectual property, and the privacy and security of donors who participate’.^5^

### 1.2 Public involvement in biobanks

Biobanks are increasingly employing public involvement and engagement strategies. Public involvement aims to have the public, patients or research participants actively contributing to the research process.^6^ Public involvement is seen as a means to produce and maintain public trust and legitimacy, which are essential for the functioning of biobanks.^6^

This increase in efforts to involve the public in processes of decision making has occurred across the health‐care sector. In the UK, ‘public involvement’ is mandated by the National Institute for Health Research and other research funders.^7^ In other national contexts, terms such as involvement and engagement are used interchangeably, but the aims of these initiatives are similar despite the linguistic variation. The Canadian Institutes of Health Research advocate ‘proactive mechanisms for dialogue and shared agenda‐setting in decisions that affect Canadians as health consumers and citizens’.^8^ In the USA, deliberative engagement strategies are commonplace, and, since 2012, the Patient‐Centered Outcomes Research Institute funds health studies that cover research questions critical to the public.^9^

### 1.3 The impact of public involvement in biobanks

Investigating the *impact* of public involvement in biobanking is an important research question. There is a growing literature on the role of public involvement in the ethical, legal and policy implications of biobanking.^10^, ^11^, ^12^, ^13^, ^14^, ^15^ In 2019, Nunn et al^6^ conducted a global review of involvement in genomic research, finding that only one third of studies involved the public and concluding that more involvement would have intrinsic value for future studies. Yet, as Nunn et al note, there are few published accounts of the impact that public involvement has had on the governance, design and conduct of biobanks,^16^ or on the public who are involved. The existing evidence on the effects, if any, of public involvement practices on biobanking, or indeed health research in general, is contested and varied.^6^, ^17^, ^18^, ^19^, ^20^ There are cases where public involvement is reported to have improved the accountability or transparency of the biobank or the recruitment and retention of participants.^21^ Others have claimed that public involvement makes no difference to research or harms it, providing legitimacy for pre‐conceived conclusions. Involvement is also criticized for ‘placating the public and speeding product development, as mechanisms for engineering consent, and as framed by narrow questions’.^22^ ^(p453)^

This review investigates the impact of public involvement on biobank studies. Due to the limited evidence in this field, we aimed to

1. Describe the impact of public involvement in biobanks, including how it is conceptualized by the study authors; in order to
2. Suggest how the research community might re‐conceptualize the impact of public involvement in biobanks.

## 2 METHODS

### 2.1 Definition of terms and scope

We based the review on INVOLVE's definitions of *the public* and *public involvement in research*, conceptualized as, ‘doing research *with* or *by* the public, rather than *to*, *about* or *for* the public’.^23^ We understand *involvement* to mean ‘mechanisms whereby there can be meaningful and legitimate public input into policy that involves dialogue between relevant publics with scientists, policy makers, and other stakeholders… [We are] not referring to unidirectional attempts to increase public awareness of certain aspects of science and technology; nor […] to the measurement of “public opinion” on certain controversial issues’.^24^ ^(p3)^

As a scoping review, we relied on the understanding and reporting of impact from study authors, rather than imposing a pre‐established definition. We thus included a wide variety of perspectives on impact, both empirical and normative.

Biobanks can be situated within a wider number of data‐intensive health research initiatives.^15^ Although the term biobank might not always be used to refer to such collections of bioresources,^25^ this term is favoured in our review since large nation‐wide projects have chosen this term before.^26^

### 2.2 Inclusion and exclusion

We included papers where the impact of public involvement in a biobank was either the primary outcome or implicitly addressed. We put no limits on publication date and excluded studies not in English, French or Spanish. We excluded viewpoints and general discussion papers, and articles with insufficient detail of the contribution of the public to the biobank. We also excluded studies of educational or awareness campaigns on biobanks; public attitudes towards biobanks and public involvement; representation and diversity of biobank participants; and ethical models of individual consent without public involvement.

### 2.3 Searching for evidence

The systematic search followed the PRISMA statement. A literature search was undertaken in January 2019 of MEDLINE, EMBASE and Web of Science databases, with the aim of identifying all peer‐reviewed journal articles published on public involvement in the biobank study process. The search strategy combined keywords within three topic domains: biobanks, public involvement and impact. Further details pertaining to the search strategy are contained in Appendix 1. We also searched the INVOLVE Evidence Library and the archives of three journals focussing on public involvement in research (*Research Involvement and Engagement, Health Expectations* and *Research for all*). A further comprehensive search of the reference lists of included studies was undertaken to identify further relevant reports of biobanks that involved the public.

### 2.4 Identifying relevant evidence

On completion of the search, titles of papers and (where available) abstracts were scrutinized for possible inclusion in the review by LLP and WK independently. Disagreements and uncertainty about eligibility were resolved through discussion until consensus was reached.

Evaluation of the impact of public involvement on biobank studies did not have to be the study authors' primary research question. We put no limits on publication date, but only studies in English, French or Spanish would be included.

### 2.5 Extracting relevant data from studies included

A data extraction table was developed. Data were extracted relating to country where the biobank operates; type and size of biobank; method and stage of public involvement; tasks addressed by involvement; description of the public actively involved; the impact of public involvement; factors that influence the impact of involvement; challenges encountered; and facilitating strategies and recommendations. While there may appear to be similarity in the categories of involvement reported, such as between ‘community engagement’ and ‘community‐based participatory research’, we recorded the methods reported by study authors.

We adapted common categories of impact found in the literature^17^ when extracting data pertaining to the impact of involvement, so as to provide a holistic definition of impact that concerned the biobank, its context and its stakeholders (eg impact on research design and delivery, and impact on researchers). Indeed, although we had initially planned to use categories already identified,^17^ after data extraction we noticed that some categories specific to the biobank, not limited to research, were needed. Consequently, we included new categories such as ‘establishment of the biobank’, ‘governance’ and ‘operations’ and we changed the name of others, for example ‘impact on the research’ became ‘impact on the biobank’. This demonstrates the importance of working iteratively when conducting scoping reviews.

LLP and WK extracted data from the papers and created categories for methods of involvement and types of impact independently, including a working definition for each category. Their tables and lists were compared: if the same category appeared in both, the category remained. If not, the authors decided through discussion whether a new category was needed, or whether the data could be included in an existing category.

### 2.6 Expanding data on studies included

For the included studies, additional grey information was sought through official websites and online searching to provide more detailed information on the context, process and impact of involvement. Such grey literature, ‘that which is produced on all levels of government, academics, business and industry in print and electronic formats, but which is not controlled by commercial publishers’,^27^, ^28^, ^29^ has been shown to be an invaluable component of any systematic review.^30^

## 3 RESULTS

### 3.1 Characteristics of studies included in the scoping review

Our search yielded 1143 records. After excluding duplicates, we screened 1030 titles and abstracts and assessed 82 full‐text articles for eligibility. Forty‐one studies met the criteria for inclusion in the review (see PRISMA Statement in Appendix 2). The publication dates for those studies ranged from 2002 to 2018.

These 41 studies covered 31 biobanks. The biobanks concerned populations from eleven countries, the United States of America (12 biobanks), the UK (7), Australia (4), Canada (3), Nigeria (2) and one in each in China, France, Germany, Italy, Japan and Kenya. The International HapMap Project operated across four countries.

Fourteen were population biobanks, 13 were disease‐specific, three were hypothetical, and one was a network of four biobanks. Also, three papers did not refer to a specific biobank, and the public involved gave input on hypothetical studies that would become actual biobanks in the future.^31^, ^32^, ^33^

More details (including the method of involvement, the tasks addressed by involvement and a short description of the public involved) of the biobanks covered in the studies included can be found in Table 1. Additionally, a Supplementary Table in Appendix 3 provides details on each biobank. This was completed with data retrieved from the grey literature.

**TABLE 1:** Overview of public involvement in biobanks in the studies included (biobanks are ordered in alphabetical order of biobank name when this was available)

Name of the biobank (when available), country	Methods	Tasks	Description of the public actively involved	References
80 biobanks in Western Australia [this group did not a formal name], Australia	Deliberative exercise	Governance	16 citizens [Most were female (n = 12), aged 45 y or older (n = 12), had post‐secondary education (n = 11), were English‐speaking only (n = 15) and self‐identified as non‐Aboriginal (n = 16), Christian (n = 8) or nonreligious (n = 8)]	Molster et al^35^
Association Française Contre les Myopathies, France	Patient‐led biobank	Governance; Drafting operating policies and procedures; Researcher approval/access (governance of specimens); Commercialization; Promotional measures & recruitment strategies; Public education; Research & future involvement ideas	Association Française Contre les Myopathies—French Muscular Dystrophy Organisation (organization of neuromuscular disease patients and their parents): 4500 members	Rabeharisoa^42^
Alaska Area Specimen Bank (AASB), United States	Community‐Based Participatory Research; Lay Advisory Panel/Community Advisory Group	Governance; Drafting operating policies and procedures; Research protocols and PISs; Researcher approval/access (governance of specimens); Promotional measures & recruitment strategies; Research & future involvement ideas	Representatives from tribal health organizations	Parkinson et al^39^
Avon Longitudinal Study of Parents and Children (ALPSAC), United Kingdom	Deliberative exercise; Focus groups; Lay Advisory Panel/Community Advisory Group; Surveys	Governance	Diverse publics, in terms of ethnicity, socio‐economic status and region	Levitt^55^
BC Biobank, Canada	Deliberative exercise	Governance	‐	Walmsley,^22^ Walmsley^50^
BC Biolibrary, Canada	Deliberative exercise	Governance; Models of consent (as distinct from reviewing the documents); Consent forms and documentation; Sample collection, storage, use and transfer; Researcher approval/access (governance of specimens); Promotional measures & recruitment strategies	25 residents of British Columbia (Demographic stratification achieved)/21 British Columbians (stratified for ethnicity, religion, occupational group, sex and level of education)	O'Doherty and Hawkins,^24^ O'Doherty et al,^56^ Burgess et al^57^
CARTaGENE, Canada	Deliberative exercise; Focus groups	Promotional measures & recruitment strategies; Overall acceptability of project (generally at start of project)	Various population segments from four Quebec regions/21 people from the 5 health regions of BC [sampling methods to ensure a diverse range of participants, life‐experiences, values and discursive styles for the deliberation]	Godard et al,^46^ O'Doherty and Burgess^58^
Centre for Alaska Native Health Research (CANHR), United States	Community‐Based Participatory Research	Governance	Local tribe councils in the villages + village representatives	Boyer et al^59^
Generation Scotland, United Kingdom	Focus groups	Governance; Sample collection, storage, use and transfer; Researcher approval/access (governance of specimens); Overall acceptability of project (generally at start of project)	10 groups were purposively sampled and chosen to reflect a range of demographics (gender, ethnicity, and age), interests (patient, voluntary and civic groups) and localities (rural or city) aiming for diversity rather than representation and differing levels of ‘felt expertise’	Haddow et al^38^
GHC/UW, United States	Focus groups; Public representative in biobank governance structure (Steering Committee)	Models of consent (as distinct from reviewing the documents); Researcher approval/access (governance of specimens); Return of results	Existing Alzheimer's cohort from research study, their surrogates and Group Health (GH) members from the Seattle, WA metro area & consumer representatives	Lemke et al^60^
Inherited Cancer Connect (ICCon) database, Australia	Focus groups; Lay Advisory Panel/Community Advisory Group	Governance; Models of consent (as distinct from reviewing the documents); Researcher approval/access (governance of specimens)	24 consumers in 3 Australian states, and from a family with a heritable cancer syndrome	Forrest et al^34^
International HapMap Project, United States, Japan, China, Nigeria	Community‐Based Participatory Research; Deliberative exercise; Focus groups; Surveys	Governance; Drafting operating policies and procedures; Researcher approval/access (governance of specimens)	Baale community leader + 1 focus group and 3 public meetings in the Yoruba community & 8 focus groups and 5 public meetings in Japan & 6 focus group and 3 public meetings with Han Chinese in China/In New York: 38 people participated in the 4 focus groups, representing a wide array of ages, ethnicities and races, and incomes. & 7‐seat Community Advisory Group & A community dialogue consisting of 3 successive sessions, with limited number of participants & More than 200 individuals attended the conference	Rotimi et al,^61^ Terry et al^37^
Kaiser Permanente, United States	Focus groups; Lay Advisory Panel/Community Advisory Group	Governance	Kaiser Permanente members in northern California, community members, participants, refusers, community panel members	Lemke et al^60^
Kilifi Genetic Birth Cohort (KGBC), Kenya	Community Engagement	Sample collection, storage, use and transfer	Chiefs [civil servants with at least 12 y of schooling, drawn from the ethnic community they serve] & Community: 8000 people attended in total, with between 50 and 300 people per meeting	Marsh et al^62^
Melbourne Genomics Health Alliance, Australia	Lay Advisory Panel/Community Advisory Group	Governance; Models of consent (as distinct from reviewing the documents); Promotional measures & recruitment strategies	‐	Watson,^63^ Melbourne Genomics Health Alliance Community Advisory Group^64^
Metastatic Breast Cancer Alliance Biobank, United States	Focus groups	Promotional measures & recruitment strategies	Patient advocacy groups convened to a think tank of stakeholders	Flowers et al^65^
Multisite keloid study, Nigeria	Community‐Based Participatory Research	Promotional measures & recruitment strategies	Keloid patients (patient advisors), community leaders, kings/chiefs	Olaitan et al^66^
NuGene, United States	Focus groups; Lay Advisory Panel/Community Advisory Group; Surveys	Consent forms and documentation; Researcher approval/access (governance of specimens)	General public in Chicago area, biorepository participants & patient advocates	Lemke et al^60^
Nottingham Health Science Biobank, United Kingdom	Lay Advisory Panel/Community Advisory Group	Governance; Models of consent (as distinct from reviewing the documents); Consent forms and documentation	5 PPI advocates, all of whom have had breast cancer or are the partners of people with a history of breast cancer	Mitchell et al,^3^ Wilcox et al^21^
Patients' Tumor Bank of Hope (PATH Biobank), Germany	Patient‐led biobank	Governance; Drafting operating policies and procedures	Breast cancer survivors	Mitchell et al^3^
Peninsula Research Bank (PRB), United Kingdom	Ad‐hoc consultation & support; Lay Advisory Panel/Community Advisory Group; Public representative in biobank governance structure (Steering Committee)	Research protocols and PISs; Researcher approval/access (governance of specimens); Return of results; Research & future involvement ideas	27 lay members (only two to six steering committee lay members attend any single meeting on a rolling basis)	Jenner et al^52^
Personalized Medicine Research Project (PMRP), United States	Ad‐hoc consultation & support; Focus groups; Lay Advisory Panel/Community Advisory Group; Public representative in ethics panel	Consent forms and documentation; Promotional measures & recruitment strategies; Public education	The two general population groups comprised 11 and 12 participants, respectively [representation across all age decades through 70+]/Adults from central Wisconsin, Marshfield Clinic employees, refusers, community representatives	Lemke et al,^60^ McCarty et al^12^
PXE International Blood and Tissue Bank, United States	Patient‐led biobank	Research & future involvement ideas	Individuals bound by the effects of mutations in the ABCC6 gene that underlies PXE	Terry et al^43^
Roswell Park Cancer Institute DataBank and Biorepository, United States	Community‐Based Participatory Research	Promotional measures & recruitment strategies	Seven members reflecting diverse demographic characteristics	Erwin et al^67^
Tasmania Biobank, Australia	Deliberative exercise	Governance; Sample collection, storage, use and transfer; Commercialization	25 Tasmanian residents of diverse backgrounds	McWhirter et al^49^
Telethon Network of Genetic Biobanks (TNGB), Italy	Ad‐hoc consultation & support; Community Engagement; Formal partnership with patient organization; Public representative in biobank governance structure (Steering Committee)	Governance; Drafting operating policies and procedures; Models of consent (as distinct from reviewing the documents); Sample collection, storage, use and transfer; Researcher approval/access (governance of specimens); Researcher approval/access (governance of specimens); Commercialization; Return of results; Incidental findings	Rare disease patient organizations & patient's associations	Filocamo et al,^40^ Baldo et al^41^
The Breast Cancer Campaign Tissue Bank (BCCTB), United Kingdom	Lay Advisory Panel/Community Advisory Group; Public representative in biobank governance structure (Steering Committee)	Governance; Researcher approval/access (governance of specimens)	Five advocates taking active roles in the tissue bank; two sit on the management board and three on the tissue access committee	Wilcox et al^21^
The Mayo Clinic Biobank, United States	Deliberative exercise; Focus groups; Lay Advisory Panel/Community Advisory Group	Governance; Drafting operating policies and procedures; Models of consent (as distinct from reviewing the documents); Research protocols and PISs; Researcher approval/access (governance of specimens); Commercialization; Return of results; Promotional measures & recruitment strategies; Research & future involvement ideas	19 members Biobank Community Advisory Board [range of ages and educational levels, half male and half female, 75% white]/20 local residents [of the Olmsted County, Minnesota community who varied by age, sex, social and economic status, race, ethnicity, and employment] in the deliberative exercise ‐ half of the participants from the deliberative exercise agreed to become members of a standing Community Advisory Board (CAB) + 10 other community members	Lemke et al,^60^ Olson et al,^36^ Kimball et al,^16^ Mitchell et al^3^
UC Biobank, United States	Deliberative exercise	Governance; Models of consent (as distinct from reviewing the documents); Sample collection, storage, use and transfer; Return of results; Public education	51 state residents as stakeholders and recruited residents from two large metropolitan areas, Los Angeles (LA) and San Francisco (SF), who had completed the 2009 California Health Interview Survey and were willing to be re‐contacted for future studies	Dry et al^68^
UK Biobank, United Kingdom	Focus groups; Lay Advisory Panel/Community Advisory Group	Promotional measures & recruitment strategies	6× 6‐8 people in the north, south and south‐west of England, in Wales and Scotland, and an additional group in north‐west England consisted of ethnic minority groups not represented in the first five [all the groups had a spread of ages, socioeconomic groups and roughly equal numbers of men and women]	Levitt and Weldon,^69^ Levitt,^55^ People Science & Policy Ltd^70^
BioVU, United States	Focus groups; Lay Advisory Panel/Community Advisory Group; Surveys	Promotional measures & recruitment strategies; Overall acceptability of project (generally at start of project)	Vanderbilt Clinic, diverse adult outpatient clinic in Nashville, TN, community members, medical centre and university faculty and staff	Lemke et al^60^
Wales Cancer Bank, United Kingdom	Ad‐hoc consultation & support	Governance; Models of consent (as distinct from reviewing the documents); Consent forms and documentation; Sample collection, storage, use and transfer; Research protocols and PISs; Promotional measures & recruitment strategies	4 lay members	Mitchell et al,^3^ NIHR Cancer Research Network (NCRN) report^71^
Not focussing on any specific biobank				Bossert et al^31^, Coors et al^32^, Lemke et al^33^

### 3.2 Methods of involvement

We identified eleven methods of involvement (listed in Table 2); many individual biobanks combined multiple methods. The most common forms were the creation of lay advisory panels (13), focus groups (13) and deliberative exercises (9). Ad‐hoc consultation and support, community‐based participatory research, public representatives in biobank governance structure and surveys were each used by four biobanks. Three were patient‐led biobanks, two used community engagement. Finally, a formal partnership with a patient organization or a public representative in an ethics panel was each reported in one biobank.

**TABLE 2:** Methods of public involvement (in alphabetical order) used in reported studies

	Method of public involvement
1	Ad‐hoc consultation & support
2	Community‐Based Participatory Research
3	Community Engagement
4	Deliberative exercise
5	Focus groups
6	Formal partnership with patient organization
7	Lay Advisory Panel/Community Advisory Group
8	Patient‐led biobank
9	Public representative in biobank governance structure (Steering Committee)
10	Public representative in ethics panel
11	Surveys

### 3.3 Aspects of biobank involvement

Biobanks invited public input into varied aspects of the organization. Most commonly, the public had a role in governance (18), and in working with researchers to determine models of consent and the design of consent forms (14). Thirteen biobanks involved the public in discussion of promotional measures and recruitment strategies. Members of the public were involved in aspects pertaining to sample collection, storage, use and transfer and data sharing in seven cases. Six biobanks involved the public in drafting operating policies and procedures and in conversations about researcher access to specimens and five in consent forms and documentation, return of results, and ideas for research and future involvement. Research protocols and participant information sheets and commercialization were a focus in four cases. Finally, three involved the public in education initiatives and in assessments of overall project acceptability, while only one involved the public in the discussions around incidental findings and IT.

### 3.4 Types of impact reported

**FIGURE 1:**
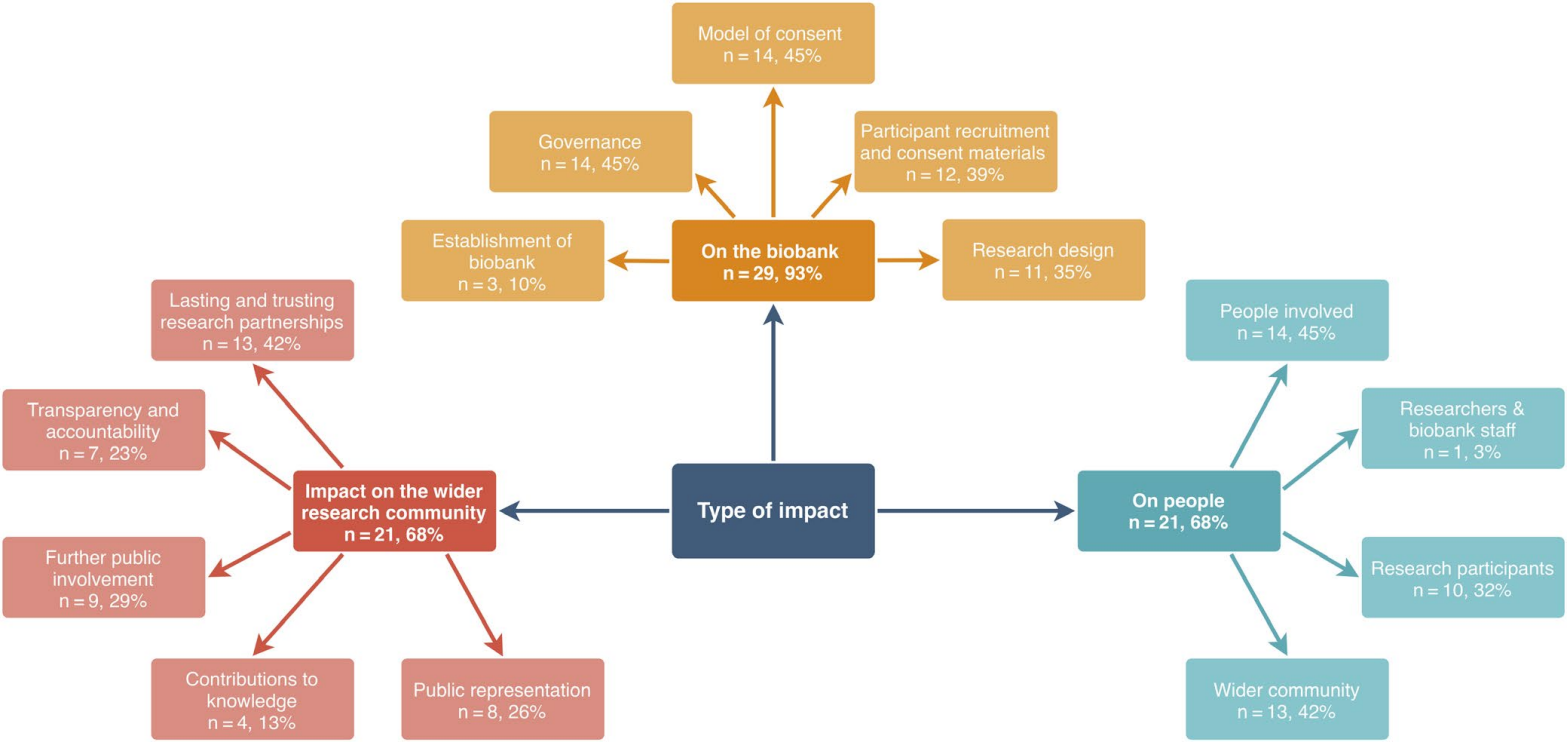
Classification of the types of impact of public involvement in biobanks (n = number of biobanks)

The impact of involvement reported in the studies included could be classified into three main categories (a detailed classification is shown in Figure 1) based on *where* the impact is seen. These were impact (a) on the biobank, (b) on the people involved and (c) on the wider research community. Within each of these categories are more precise locations (detailed in Appendix 4).

In the first category (impact on biobank), the most common forms of impact reported were changes to the models of consent and the design of consent forms (n = 14). Other common forms of impact were new policies and regulations and new recruitment strategies and materials (n = 12). Secondly, regarding impact on people, a common form of impact was education of communities (n = 10). In the third category, the most common impacts to the wider research community reported were claims of some form of lasting and trusting research partnership (n = 14) and the new issues raised by members of the public involved (n = 12). Also, nine of the biobanks reported that they decided to expand their public involvement activities.

Of the forty‐one studies included, less than one‐third (n = 12) included members of the public as authors, or in the reporting of impact (eg through quotes).

Twenty‐one biobanks also made normative claims (ie value judgements about impact, as opposed to descriptive claims) about the impact of public involvement. In these cases, authors reported that involving the public led to increased transparency, accountability, trust and lasting research partnerships, but they did not provide evidence.

#### 3.4.1 Impact on the biobank

##### Impact on model of consent (n = 14)

Biobanks that had their models of consent shaped by the public either (a) give a public group a model of consent to review or (b) run a deliberative exercise to generate principles that should appear in a model. Forrest et al^34^ provides an example of the former, where the public commented on a pre‐designed model of consent. Nominal group techniques were used during public forums, to gather qualitative data on participants' attitudes towards the establishment of a national research database.^34^ Molster et al^35^ provides an example of the second approach, where guidelines written with members of the public recommended that transparency, autonomy and good communication should be at the heart of the consent process. Those involved also produced a list of topics to be communicated to potential biobanking participants as part of an informed consent process.

##### Impact on participant recruitment and consent materials (n = 12)

Studies reporting the impact of public involvement on the production of participant materials and consent forms typically involved simplifying the language and shortening recruitment materials. Kimball et al^16^ illustrates this process in the Mayo Clinic Biobank, where the Community Advisory Board produced a series of recommendations to improve recruitment and consent materials. Their suggestions, of which several were implemented, included shortening recruitment materials and modifying language on informed consent documents.

##### Impact on governance (n = 18)

Of biobanks that involved the public in governance matters, two‐thirds (n = 12) reported new guidelines and policies as outcomes of involvement. Reporting varied and authors provided different levels of detail. Often the impact was not clear since ‘governance’ labelled many different practices and processes. It was particularly difficult to determine the impact of involvement when the public were embedded into a governance process. However, some authors provided examples of deliberative exercises that led to the recommendation of specific policies.

In most cases, the impact of public involvement on governance is vague. For example, the deliberation exercise in O'Doherty and Hawkins^24^ on the ‘Governance of Biospecimens and Associated Data’ reported only that the personnel of the biobank had obtained ‘valuable input’ from attendees, which they could use to set up the governance structure of the biobank. Olson et al^36^ reports a 4‐day deliberative community exercise during which attendees reviewed Mayo Clinic Biobank policies, before making recommendations. They suggested guiding principles around biobank procedures including ‘the need for strong privacy protections, convenient recruitment, the importance of data sharing, limited options for return of research results, the importance of long‐term community oversight, and an easy‐to‐understand consent document’. However, the authors do not provide information on how those were implemented.

Finally, in a few cases authors discussed exercises to develop national biobank policy, rather than involvement in a specific biobank. Terry et al^37^ reports on dialogue sessions that developed policy recommendations, with the recommendations summarized in a table including details on each. These new policies were later presented in a forum for researchers and the public, and then to the wider community. Molster et al^35^ describes a deliberative exercise in Australia, aimed at understanding ‘citizen perspectives, shared values and acceptable trade‐offs in public interests’. During the event 16 deliberants formulated 28 recommendations around broad areas (Rules and regulations, Oversight, Biobank participation, Access and use, Information, Benefit‐sharing and Demise [ie study end; for example, ‘research samples and data should be destroyed after the completion of the yes research’^35^]). These recommendations were contrasted with existent policy, and authors note that ‘most of the deliberants' overarching principles, issues of importance and recommendations were reflected in the policy’. For most of the recommendations that were not yet reflected in policy, ‘experts’ translated them into biobanking guidelines. The authors acknowledge that ‘a minority of deliberants' recommendations were not incorporated in the policy’ and provide reasons. Finally, they were published as guidelines to be adhered to by the Western Australia government health agencies.

#### 3.4.2 Impact on people

We identified four main groups to which the studies referenced impact: (a) members of the public ‘involved’, (b) researchers and biobank staff, (c) research participants and (d) the wider community. The wider community was the group most often discussed, as around a third (n = 10) of biobanks reported education of the wider community as an impact and eight biobanks reported ‘wider engagement of the community with science and research’. In a few cases, public involvement appeared to be a two‐way learning process, with nine biobanks noting that researchers gained awareness of needs and expectations of the public. Involvement had an impact on trust, education, skills and other personal aspects in five biobanks. The impact on those involved is mostly vague, as members of the public are rarely involved in reporting impact. Also, some authors highlighted impact on wider biobank participants (n = 7). Finally, ‘further involvement’ was cited as an impact on those involved in nine biobanks. For example, one of the attendees at a deliberation exercise would join the Governance Oversight Committee.^24^

Very few biobanks reported any negative impact on the members of the public involved, despite studies outside of this review arguing that the complexity of topics discussed and lack of clarity of process can lead to dissatisfaction and frustration with the process of public involvement.^38^

#### 3.4.3 Impact on the wider research community

##### Impact on further public involvement (n = 9)

For nine biobanks, an impact of public involvement was to develop further public involvement initiatives, by the same or other members of the public. This happened in four distinct forms: (a) one‐off involvement became a sustained form (eg the creation of Advisory Groups to ensure public perspectives is shared at all stages of the biobank), (b) involvement started with a small group of people and was then formalized through patient organizations to create a community with wider representation and to optimize resources for research on rare conditions, (c) in user‐led biobanks for rare diseases, users pursued wider collective action to advocate for research and treatment of those affected and (d) new questions to be explored in the future was raised by those involved.

In three cases,^3^, ^33^, ^39^ initial involvement led to the establishment of lay advisory boards. In the Wales Cancer Bank, an initial steering group composed of 28 stakeholders, of which three were patients and one a relative of patient, evolved into a larger Lay Liaison and Ethics group (LLEG).^3^ The Chair is a full member of the Executive group, so that public voice has progressively become an integral part of biobank governance structures. For the Mayo Clinic, a Community Advisory Board (CAB) was created with the aim of keeping sight of community interests. This initiative followed recommendations from a community engagement event ‘to facilitate community influence in the development and governance of the biobank’^33^ at its early stages.^3^, ^33^

In other cases, some forms of involvement led to more opportunities for involvement through formal agreements. In the case of the Telethon Network of Genetic Biobanks (TNGB), the involvement of patients and families led to the formalization of various agreements between the TNGB and patients' organizations (PO).^40^ Opportunities for dialogue with the public (34 events) were promoted through POs.^41^ In these arrangements, POs have multiple roles: they must (a) select a representative that updates associated families and referring clinicians on the biobank's activities and policies; (b) promote the recruitment of patients and relatives, and (c) organize shipment of biospecimens to the assigned biobank.^41^ Successful involvement of POs led to formal collaboration with other POs, resulting in a centralized catalogue of very unique samples and ‘sustained infrastructure’ that encouraged more research into those ‘neglected’ diseases.^40^

##### Lasting and trusting research partnerships (n = 13)

When working with patients' organizations and advocacy groups, study authors often claimed ‘lasting or trusting research partnerships’ as forms of impact. These are commonly found in the literature and were considered here as examples of normative claims.

Public involvement in rare disease biobanks has led to impact on the wider research community. For example, what Rabeharisoa reported as an early example of Pos' impact on biobanking.^42^ In the case of the *Association Française Contre les Myopathies* (AFM), self‐help and advocacy movements converged in their aim for ‘users' empowerment’ and illustrated the legitimacy and the ability of POs to organize collective action advocating research and care for rare diseases. The AFM, through its unique biobank, also achieved a definition of the disease and its status. In addition, POs can have an impact on other advocacy groups focusing on different rare diseases. For example, the AFM led the creation of the *Alliance Française des Maladies Rares* (French Alliance for Rare Diseases), an umbrella organization currently grouping together 80 patient organizations. Similarly, Terry provides an example of how patients and their families formed a biobank for research on a particular disease.^43^ In this case, those affected by Pseudoxanthoma elasticum (PXE) and their families formed a community that could represent their needs and, in response, those affected by PXE came forward to donate their samples. Success in the creation of the biobank encouraged the community to lead other PXE research projects, as bonds strengthened between patient community and researchers. Moreover, they have since mentored other advocacy groups and created the Genetic Alliance Biobank, a coalition of over 600 disease advocacy organizations, that uses PXE's biobank infrastructure as a repository for their model and methods.

Finally, in twelve cases, the public raised new research questions. For example, Kimball et al^16^ provides a list of the questions raised by members of the community advisory board, including the value of genomic research and its clinical utility; the risk of genetic discrimination; and personal ownership of genomic data and the distinction between indirect benefits to future generations and individual risk to research participants.

## 4 DISCUSSION

### 4.1 Summary

This review included forty‐one studies covering thirty‐one biobanks from eleven different countries. Three main findings can be highlighted.

Firstly, our review illustrates the broad range of issues and topics addressed through a wide range of involvement methods, as Nunn et al^6^ have reported. Studies used varying degrees of active roles for members of the public, and in almost half of the cases, combined up to four methods of involvement. Amongst these, the most frequent were advisory groups, deliberative exercises and focus groups. This could be due to both a lack of clarity of purpose in why public involvement is being undertaken in a biobanking context, and varying conceptualizations of what it should look like.

**FIGURE 2:**
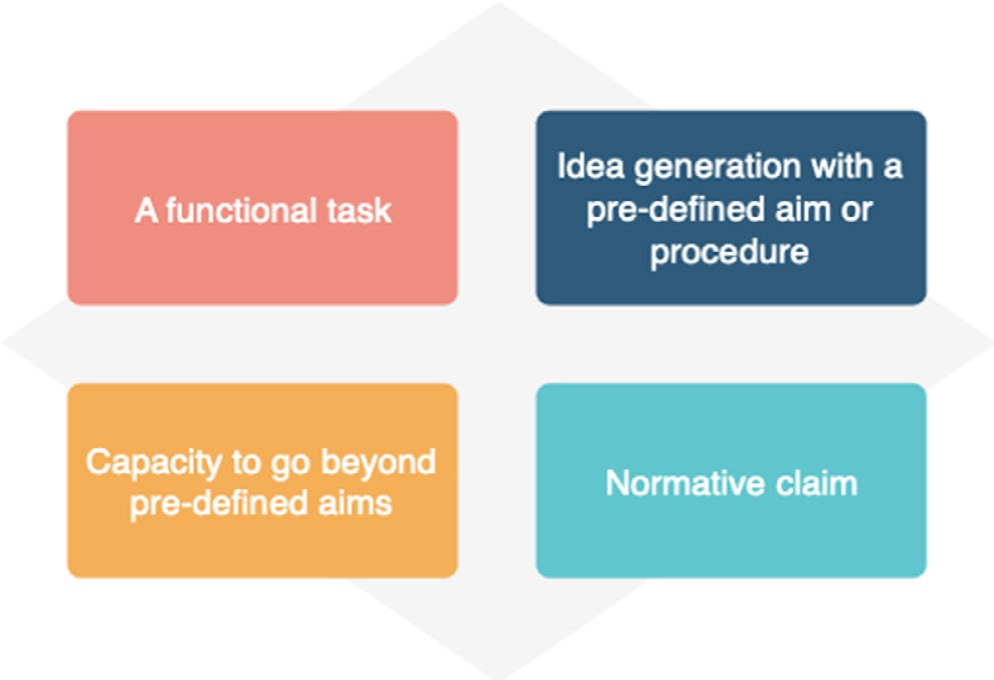
Extent of reported impact of public involvement in biobank

Secondly, most studies focused on functional involvement that worked to improve the efficiency of existing biobank activities. This can be characterized as using participation to achieve predetermined project goals and objectives. The wider literature on public participation has recognized this dynamic in other projects, where ‘such involvement may be interactive… but tends to arise only after major decisions have already been made by external agents’.^44^ Based on the studies included in this review, we suggest that the extent of the reported impact involvement in biobanks can be conceptualized in four broad types (Figure 2). The most cited form of involvement is the public performing a functional task, mostly with the purpose of improving participation rates. Accordingly, the impact centres around the enhancement of consent forms, processes and recruitment. In other instances, the public were involved in exercises of idea generation but within a pre‐defined aim or procedure. Also, more than a third of the studies included made normative claims about the impact of involvement but did not include evidence to support these.

Thirdly, only in a few cases did the public's influence go beyond the aims pre‐defined by the biobank. Although there were cases where a broader form of ‘impact’ was seen through involvement, this type of impact was only vaguely defined and reported. This supports the first and second findings—that there is a lack of clarity over purpose in conducting involvement beyond improving participation rates. It also suggests either a lack of consideration of impact or a lack of understanding of how to conceptualize it.

### 4.2 Strengths and limitations of our review

To our knowledge, this is the first attempt to review the *impact* of public involvement in biobanking. This review provides a narrative summary of how impact has been captured and conceptualized, before discussing some of the difficulties encountered. Our results are consistent with previous studies exploring methods of involvement in biobanks which suggest the dominance of functional approaches. Unlike previous studies, our review departs from attempts to quantify ‘outcomes’ of public involvement and turns to issues associated with the conceptualization of impact by researchers.

Our study had several limitations. The level of detail when reporting impact varied considerably, partly due to the challenges of capturing it. Bossert et al^31^ used a traffic‐light system to track changes to materials introduced by members of the public involved. The Wales Cancer Bank includes in its annual reports the projects undertaken and features written by its lay collaborators so as demonstrate the value of public involvement within the biobanks.^3^ Due to the lack of details on the impact reported, we could not explore the influence of contextual and process factors such as the method of involvement, the length of the intervention or partnership, or the quality of relationships between researchers and the public. One way to solve this issue would be for authors to adhere to the Standardized Data on Initiatives—(STARDIT) initiative,^45^ which proposes a standardized way of describing the ‘who’, ‘how’ and ‘what’ of initiatives such as research as well as a model for capturing two‐way learning and ‘transformational learning’ amongst other impacts.

Also, in line with finding of this review which suggested most impacts were likely not reported,^6^ another limitation of our study is that details about public involvement in most of the biobanks included came from peer‐review literature. Inclusion of more and wider methods of reporting of impact, which we attempted by including grey literature, might have provided further details. Also, to remain consistent in face of this limitation, we focused on how biobank authors interpret impact, rather than aiming to demonstrate impact ourselves.

Moreover, since evaluation does not include long‐term follow up, we could not explore some of the impacts to the wider research community, such as national policies emerging from the public's recommendations or greater trust of the community towards biobanks. Finally, our study can only account for forms of impact that are reported in the literature, limited in most cases to the perspective of experts.

We pose three areas for further research and consideration by biobank researchers and public involvement practitioners.

#### 4.2.1 Biobanks' functional approach to public involvement limits impact

This review demonstrates that many biobanks pursue public involvement with a functional objective of increasing participation. A functional approach to involvement leads to largely functional outcomes. Biobanks that choose such an approach to public involvement encounter two major risks.

Firstly, this approach can mislead the public about their potential influence. Clarity when inviting involvement^20^, ^46^ will prevent the obfuscation of goals and subsequent public frustration. This recommendation echoes Lemke's suggestion that ‘biobank community engagement efforts need to have clearly defined goals’.^33^ If involvement is perceived by the public simply as another tool for researchers to achieve participation in their studies, biobanks face a ‘risk that the public will mistrust researchers and will simply not participate in sufficient numbers’.^47^

Secondly, a narrow scope for the public limits involvement to existing objectives; people's needs, values and concerns (including vulnerable people such as indigenous groups^48^) cannot then shape the study. There is some evidence that the ‘involved’ public wish to move beyond pre‐defined roles set for them by the biobank, as they raised additional questions that they considered important. Terry et al^37^ highlights that ‘participants in our project wanted fair and equitable access, and wanted a voice in the process’ which led to expansion beyond the topics suggested by researchers. Researchers and biobank managers may be stretched and challenged constructively beyond the limits of established processes by enabling the generation of new questions and ideas. Indeed, this ‘[f]lexibility is required to ensure that participants are able to express the values they feel are most relevant to the issue. In imposing structure on deliberation, the event designers may have gotten it wrong, and a degree of willingness to be guided by participants is essential’.^24^ (see also Ref.^21^, ^37^). Assessment is facilitated by asking from the beginning, ‘Why are we involving the public?’

#### 4.2.2 Disagreement in public involvement is valuable and should be captured

Public involvement is often presented as a process that is neutral and technical. But it has too an essentially political nature^49^ and, particularly under the form of public deliberation, often results in persistent disagreement,^50^ which creates an inherent tension in trying to achieve involvement that is meaningful to all stakeholders. While some studies included in this review referred to certain tensions and moments of disagreement,^35^ these are sparse and poorly acknowledged. Reporting of involvement by study authors is often limited to outcomes from the research defined prior to involvement.

Walmsley^51^ highlights the dangers of deliberation methodologies that focus solely on consensus, arguing that it is important to ensure that conflict is possible within deliberation spaces. Her study advocates for persistent disagreement as an output of involvement, and she writes, ‘we need to develop innovative ways of reporting agonistic deliberation as well as consensus—recording the frustrations, road‐blocks, contested definitions and repeated questions that hamper attempts to reach “recommendations” and “outputs” as traditionally conceived’.^21^

Calls within the wider involvement literature are made for researchers to ‘receive constructive criticisms and engage in constructive conflict’.^52^ Practical suggestions by authors in the review included Kimball et al^16^ who advises a semi‐structured guide to ensure that the agenda can leave room for new questions brought by members of the public. Jenner et al^53^ included a ‘lay member ideas’ section in the agenda, as an ‘open forum’ where members could bring their own concerns and interests to the table. This type of initiative was suggested by Lemke to build ‘a relationship of mutual learning and trust’^11^ (see also Ref.^12^).

#### 4.2.3 The impact of public involvement needs to be re‐imagined

This study has demonstrated the inherent difficulties in capturing the impact of public involvement. Firstly, the lack of clarity over impact arises because of the challenges of defining ‘impact’^17^, ^18^, ^19^, ^54^ and competing rationales for its investigation. To some, public involvement should be able to produce a demonstrable change in the research to justify its existence, much like an intervention. To others, impact is better conceived as a process of reflective learning between researchers and the public. Some outcomes are more readily quantifiable, such as improved participation rates, while others are highly subjective or unpredictable, such as changing researcher attitudes. Consequently, it is difficult to expect a uniform standard for conducting and evaluating public involvement activity.

Secondly, the biobank studies included were often written by researchers and thus considered impact from the perspective of the biobank and experts. This has serious limitations for determining the impact, if any, that involvement had on the public's involved directly or indirectly. It is likely that different groups have different priorities. For example, while impact for some researchers may represent improved recruitment rates, patients may prioritize outcomes that ‘matter to them and their communities’.^46^ ^(p201)^ Without public involvement, it is difficult to answer a question such as the *effect on what and for whom?*

Thirdly, beyond the largely functional aspects of public involvement, many biobanks made normative claims that public involvement led to increased transparency, accountability and lasting, trusting research partnerships. These claims appear to be more difficult to evidence than the impact of task‐based involvement, because they require different methods of design and analysis over a longer period.

This study supports a growing call in the literature for an approach that conceptualizes involvement as *conversations that support two‐way learning* ^52^ (see also Ref.^55^). This is an approach for both biobanking and wider health and social care research. Less focus on mandating the reporting of quantifiable outcomes will enable greater focus on the process of reflective learning for researchers and the public in partnership. Organizing and facilitating involvement in biobanks consumes time and resources, and consideration of best practices and guidance is important. Researchers might currently find it difficult to evidence ‘non‐functionalist’ forms of impact or might lack awareness of its relevance. Alongside an approach focused on reciprocity, funders should also support longer‐term, social science research to understand varieties of ‘public involvement’. Methods to capture more subjective forms of impact need to be developed to improve reflective two‐way learning.

## 5 CONCLUSIONS

The functional approach to public involvement reported from most biobanks limits likely impact. Reporting of involvement by study authors is often limited to outcomes from the agenda of researchers defined prior to involvement. This conceptualization of involvement emerges from long‐standing disagreement about why public involvement is valuable, and an entrenched neutral and technical understanding that ignores the political nature of involvement.

There are several inherent difficulties in trying to capture impact, both epistemological and methodological, not least the competing rationales for why impact should be investigated. Ultimately, this study urges a re‐imagination of impact, re‐conceptualized as a two‐way learning process. More support must be provided to researchers and the public to undergo such reflective exercises.

## CONFLICT OF INTEREST

There were no financial support or other benefits from commercial sources for the work reported on in the manuscript, or any other financial interests that any of the authors may have, which could create a potential conflict of interest or the appearance of a conflict of interest with regard to the work.

## Data Availability

The data that support the findings of this study are available from the corresponding author upon reasonable request.

## Appendix 1 Search strategy details

**Table:**

	Search domain	Search terms
1	BioBank	biobank*.mp OR exp biobank as Topic/
2	Public involvement	((consumer* or citizen* or client* or carer* or communit* or lay or patient* or public* or service user* or user* or survivor* or stakeholder* or family or families or relative* or parent*) and (involv* or collaborat* or engage* or partner* or consult* or advis* or emancipat* or empower* or advocat* or embed* or represent* or particip* or led)).ti.
3	Public involvement outcomes	(impact* or effect* or adapt* or modif* or change* or develop* or design* or improve* or worse* or increase* or boost* or decreas* or reduc* or differ* or edit* or suggest*).ab,ti
4	1 and 2 and 3	

## Appendix 3 Details of biobanks covered in the studies included

**Table:**

Name of the biobank (when available), country	Date established	Size	Type of biobank	Host organization	Country or region	References
80 biobanks in Western Australia [this group did not a formal name], Australia	proposed biobank	Not available	Disease‐specific &. Population biobank	Department of Health WA	Perth, Western Australia, Australia	Molster et al^35^
Association Française contre les Myopathies, France	1958	Not available	Disease‐specific biobank	AFM (Association Francaise contre les Myopathies) financed by Telethon	France	Rabeharisoa^42^
Alaska Area Specimen Bank (AASB), United States	1961	266 353 residual biologic specimens (serum, plasma, whole blood, tissue, bacterial cultures) from 83 841 persons who participated in research studies	Population biobank	Alaska Natives (Indian Self Determination Act (Public Law 93‐638 (1975))	Alaska, USA	Parkinson et al^39^
Avon Longitudinal Study of Parents and Children (ALPSAC), United Kingdom	1991‐2	Not available	Population biobank	University of Bristol (Medical Research Council, the Wellcome Trust)	Bristol/Bath, UK	Levitt^56^
BC Biobank, Canada	2007	Not available	Population biobank	University Of British Columbia Department of Pathology and Laboratory Medicine, the Canadian Tissue Repository Network and the BC Cancer Agency	British Columbia, Canada	Walmsley,^22^ Walmsley^51^
BC Biolibrary, Canada	2007	Not available	not a biobank, a network to integrate and improve access and quality of several provincial biobanks	University of British Columbia	British Columbia, Canada	O'Doherty and Hawkins,^24^ O'Doherty et al,^57^ Secko, ^73^
CARTaGENE, Canada	Not available	aims to recruit a random sample of 50 000 individuals between the age of 25 and 75	Population biobank	Sainte‐Justine Children's Hospital University Health Center	Quebec, Canada	Godard et al,^47^ O'Doherty and Burgess^59^
Centre for Alaska Native Health Research (CANHR), United States	Not available	30% of eligible individuals (5000 eligible?)	Population biobank	Centre for Alaska Native Health Research (CANHR)	Canada	Boyer et al^60^
Generation Scotland, United Kingdom	Not available	large family‐based cohort study (recruiting from age group 35‐55) with the eventual goal of collecting blood for the purpose of extracting DNA from 50 000 individuals	Population biobank	Partnership ‐ four Scottish universities & NHS Scotland	Scotland, UK	Haddow^38^
GHC/UW, United States	1994	1200 living participants; 1700 deceased	Disease‐specific biobank	eMERGE Network ‐ National Human Genome Research Institute (NHGRI)	Seattle area, Washington, USA	Lemke et al^61^
Inherited Cancer Connect (ICCon) database, Australia	2013	Not available	Disease‐specific biobank	Inherited Cancer Connect (ICCon) Partnership ‐ Cancer Council of New South Wales, Australia	Australia	Forrest et al^34^
International HapMap Project, United States, Japan, China, Nigeria	since 1980s	Not available	Population biobank	International HapMap Consortium	USA, Japan, China, Nigeria	Rotimi et al,^61^ Terry et al^37^
Kaiser Permanente, United States	2005	All diseases represented in patient population – 160 000	Population biobank	eMERGE Network ‐ National Human Genome Research Institute (NHGRI)	Northern California, USA	Lemke et al^61^
Kilifi Genetic Birth Cohort (KGBC), Kenya	1989	aims to recruit 12 000 infants	Disease‐specific biobank	Kenya Medical Research Institute (KEMRI) ‐ Wellcome Trust Research Programme + MalariaGEN Consortium	Kenya	Marsh et al^63^
Melbourne Genomics Health Alliance, Australia	2014	Not available	Population biobank	Partnership ‐ 10 research organizations & hospitals	Melbourne, Australia	Watson^63^‐ Melbourne Genomics Health Alliance Community Advisory Group^65^
Metastatic Breast Cancer Alliance Biobank, United States	proposed biobank	Not available	Disease‐specific biobank	Metastatic Breast Cancer Alliance	USA	Flowers et al^66^
Multisite keloid study, Nigeria	2005	4200 samples from participants from 103 families	Disease‐specific biobank	University of Connecticut Health Center (UCHC) General Clinical Research Center (GCRC) & LAUTECH in Osogbo and UCH in Ibadan	Ibadan (Oyo State) and Osogbo (Osun State), Nigeria	Olaitan et al^67^
NUgene, United States	2001	All diseases represented in patient population	Population biobank	Northwestern University ‐ University medical centre ‐ eMERGE Network ‐ National Human Genome Research Institute (NHGRI)	Chicago area, Illinois, US	Lemke et al^61^
Nottingham Health Science Biobank, United Kingdom	2011	The aim is to consent every patient at the time of first presentation at our hospital and those referred for surgery and treatment after diagnosis (ongoing)	Population biobank	Nottingham University NHS Hospitals Trust	Nottingham, UK	Mitchell et al,^3^ Wilcox et al^21^
Patients' Tumor Bank of Hope (PATH Biobank), Germany	2002	Approximately 7500 patients have consented to be donors with seven sample source sites across Germany (ongoing)	Disease‐specific biobank	PATH Foundation, Germany	Germany	Mitchell et al^3^
Peninsula Research Bank (PRB), United Kingdom	Not available	Not available	Population biobank	PenCLARHC: Peninsula Collaboration for Leadership in Applied Health Research and Care ‐ NIHR Exeter Clinical Research Facility (CRF)	Exeter, UK	Jenner et al^52^
Personalized Medicine Research Project (PMRP), United States	2002	All diseases represented in patient population – 20 000	Population biobank	Marshfield Clinic ‐ Medical centre ‐ eMERGE Network ‐ National Human Genome Research Institute (NHGRI)	Central Wisconsin, USA	Lemke et al,^61^ McCarty et al^12^
PXE International Blood and Tissue Bank, United States	1995	Not available	Disease‐specific biobank	PXE International	USA	Terry et al^43^
Roswell Park Cancer Institute DataBank and Biorepository, United States	Not available	Not available	Disease‐specific biobank	Roswell Park Cancer Institute Alliance Foundation and NIH ‐ National Cancer Institute (NCI)	Niagara Falls, NY, USA	Erwin et al^68^
Tasmania Biobank, Australia	hypothesized biobank	Not available	Not available	Not available	Tasmania, Australia	McWhirter et al,^50^ Chalmers et al^4^
Telethon Network of Genetic Biobanks (TNGB), Italy	2008	90 000 biological samples representing approximately 850 distinct rare genetic diseases	Disease‐specific biobank	a consortium of 11 Italian non‐profits supported by the Telethon Foundation	Italy	Filocamo et al,^40^ Baldo et al^41^
The Breast Cancer Campaign Tissue Bank (BCCTB), United Kingdom	2010	8230 samples	Disease‐specific biobank	Breast Cancer Now and 5 UK universities	UK	Wilcox et al^21^
The Mayo Clinic Biobank, United States	2009	The target goal is to obtain 50 000 biospecimens (ongoing)	Population biobank	Mayo Clinic, Rochester	Minnesota, USA	Lemke et al,^60^ Olson et al,^36^ Kimball et al,^16^ Mitchell et al^3^
UC Biobank, United States	hypothesized biobank	Not available	Not available	EngageUC (UC Davis, UC Irvine, UC Los Angeles, UC San Diego, and UC San Francisco)	USA	Dry et al^68^
UK Biobank, United Kingdom	2000	aims to enroll 500 000 participants aged 45‐69 for a period of several year	Population biobank	MRC/Wellcome Trust	UK	Levitt and Weldon,^70^ Levitt,^56^ People Science & Policy Ltd^71^
BioVU, United States	2007	All diseases represented in patient population	Population biobank	eMERGE Network ‐ National Human Genome Research Institute (NHGRI)	Tennessee, USA	Lemke et al^61^
Wales Cancer Bank, United Kingdom	2004	Currently 12 000 patients (ongoing)	Disease‐specific biobank	Cardiff University	Wales, UK	Mitchell et al,^3^ NIHR Cancer Research Network (NCRN) report^72^
						Bossert et al^31^; Coors et al^32^; Lemke et al^33^

## Appendix 4 Details of the impacts of public involvement on each biobank

**Table:**

Impact type	Biobanks	Papers demonstrating this impact	Example
On Biobank			
Governance			
Written agreement with patient organizations	AFM; Telethon Network of Genetic Biobanks	Baldo et al,^41^ Filocamo et al,^40^ Terry et al,^43^ Rabeharisoa^42^	‘interest on the part of Patient Organisations in the biobanking service, which has led to 13 written agreements designed to formalise this process. These agreements enabled the centralisation of rare genetic disease biospecimens and their related data’ (Baldo et al^41^)
New policies and regulations	80 biobanks in Western Australia; AFM; Alaska Area Specimen Bank (AASB); BC Biolibrary; CARTaGENE; International HapMap Project; Mayo Clinic Biobank; Metastatic Breast Cancer Alliance Biobank; Tasmania Biobank; BCCTB; Telethon Network of Genetic Biobanks; UC Biobank	Baldo et al,^41^ Dry et al,^69^ Filocamo et al,^40^ Flowers et al,^66^ Molster et al,^35^ O'Doherty and Hawkins,^24^ O'Doherty et al,^57^ Olson et al,^36^ Parkinson et al,^39^ Secko, 2008, Terry et al,^37^ Wilcox et al,^21^ O'Doherty and Burgess,^59^ McWhirter et al,^50^ Lemke et al,^61^ Terry et al,^43^ Rabeharisoa^42^	during a deliberative exercise 16 deliberants formulated 28 recommendations around broad areas (Rules and regulations, Oversight, Biobank participation, Access and use, Information, Benefit‐sharing and Demise) that experts took onboard and translated into biobanking guidelines (Molster et al^35^)
Standardization of procedures across biobanks	AFM; CARTaGENE; Melbourne Genomics Health Alliance; PXE	O'Doherty and Burgess,^59^ Lemke et al,^61^ Watson,^64^ Terry et al,^43^ Rabeharisoa^42^	‘call for independent governance of biobanks and call for standardization of procedures within and between biobank’ (O'Doherty and Burgess^59^)
Centralization of biospecimens	BCCTB; Telethon Network of Genetic Biobanks	Baldo et al,^41^ Wilcox et al^21^	agreements with 13 patient organisations enabled the centralisation of rare genetic disease biospecimens and their related data (Baldo et al^41^)
Agreement to share resources	BCCTB; Telethon Network of Genetic Biobanks	Baldo et al,^41^ Filocamo et al,^40^ Wilcox et al^21^	‘give the Network of biobanks access to a critical mass of samples essential for research’ (Filocamo et al^40^)
Operations			
Models of consent and informed consent forms	80 biobanks in Western Australia; BC Biolibrary; BCCTB; ICCon; Mayo Clinic Biobank; Melbourne Genomics Health Alliance; NuGene; Nottingham Health Science Biobank; Personalized Medicine Research Project (PMRP);Tasmania Biobank; Telethon Network of Genetic Biobanks; The Peninsula Research Bank; UC Biobank; Wales Cancer Bank	Baldo et al,^41^ Bossert et al,^31^ Dry et al,^69^ Filocamo et al,^40^ Forrest et al,^34^ Kimball et al,^16^ McCarty et al,^12^ Mitchell et al,^3^ Molster et al,^35^ O'Doherty et al,^57^ Olson et al^36^; Wilcox et al,^21^ McWhirter et al,^50^ Jenner et al,^53^ Lemke et al,^61^ Watson,^63^ NIHR Cancer Research Network (NCRN)^72^	‘draft of comprehensive informed consent that became the official model adopted by all the biobanks in the Network’ (Baldo et al^41^)
Recruitment strategies and materials (including consent)	BC Biolibrary; BCCTB; BioVU; CARTaGENE; International HapMap Project; Kaiser Permanente; Mayo Clinic Biobank; Multisite keloid study; Nottingham Health Science Biobank; PXE; Tasmania Biobank; Wales Cancer Bank	Bossert et al,^31^ Chalmers et al,^4^ Coors et al,^32^ Godard et al,^47^ Kimball et al,^16^ Mitchell et al,^3^ O'Doherty et al,^57^ Olaitan et al,^67^ Olson et al,^36^ Rotimi et al,^62^ Terry et al,^37^ Terry et al,^43^ Wilcox et al,^21^ Lemke et al,^61^ NIHR Cancer Research Network (NCRN)^72^	feedback was used to revise the recruitment and consent materials: eg shorten the recruitment documents to make them more concise and easier to digest (Kimball et al^16^)
Support for members of the public (hotlines, newsletters)	Mayo Clinic Biobank; Melbourne Genomics Health Alliance; Personalized Medicine Research Project (PMRP); The Peninsula Research Bank	Kimball et al,^16^ McCarty et al,^12^ Mitchell et al,^3^ Jenner et al,^52^ Lemke et al,^60^ Watson^63^	recommended the ‘publication of a community newsletter and website to inform participants and community members about the biobank’ (The Mayo Clinic Biobank, Lemke et al^60^)
Access to specimens/data	AFM; Alaska Area Specimen Bank (AASB); BCCTB; Nottingham Health Science Biobank; PXE; Telethon Network of Genetic Biobanks; UK Biobank; Wales Cancer Bank	Baldo et al,^41^ Boyer et al,^60^ Filocamo et al,^40^ Levitt and Weldon,^70^ Mitchell et al,^3^ Terry et al,^43^ Wilcox et al,^21^ Rabeharisoa,^42^ NIHR Cancer Research Network (NCRN)^71^	‘the level of confidence established for both donors and professionals is shown by the very high level of donation of tissue to the Bank’ (NCRN)
Confidentiality	Telethon Network of Genetic Biobanks; BC Biolibrary; Tasmania Biobank; BCCTB; Mayo Clinic Biobank	Baldo et al,^41^ Filocamo et al,^40^ O'Doherty et al,^57^ Olson et al,^36^ Wilcox et al,^21^ McWhirter et al^49^	set of rules established ensured ‘individuals' confidentiality throughout the entire process’ (Baldo et al^41^)
Increased participation & retention	Alaska Area Specimen Bank (AASB); Avon Longitudinal Study of Parents and Children (ALPSAC); BCCTB; Melbourne Genomics Health Alliance; UK Biobank	Boyer et al,^60^ Levitt,^56^ Wilcox et al,^21^ Watson^64^	‘inspired loyalty from a much larger group as demonstrated by the high participation rates’ (Levitt^56^)
Recommendations for return of research results	Mayo Clinic Biobank; Nottingham Health Science Biobank; PATH; Personalized Medicine Research Project (PMRP); Tasmania Biobank	Coors et al,^32^ McCarty et al,^12^ Mitchell et al,^3^ Olson et al,^36^ Wilcox et al,^21^ McWhirter et al,^50^ Lemke et al^61^	‘strong support for a Tasmanian Biobank and their deliberations resulted in specific proposals in relation to […] return of results’ [eg ‘A secure key should be available to reconnect identification data and samples’] (McWhirter et al^50^)
Recommendations for sharing research results	BCCTB; CARTaGENE; Kaiser Permanente; Mayo Clinic Biobank; Nottingham Health Science Biobank; NuGene; PATH; Tasmania Biobank; UK Biobank	Godard et al,^47^ Levitt and Weldon,^70^ Mitchell et al,^3^ Olson et al,^36^ Wilcox et al,^21^ McWhirter et al,^50^ Lemke et al^61^	‘11 key recommendations, outlining […] the need to offer aggregate—and where appropriate, individual—research findings to participants; and the clear description of data sharing plans as part of the informed consent process’ (Lemke et al^61^)
Funding	Melbourne Genomics Health Alliance	Watson^64^	during 2014 Victoria local election campaign, CAG helped lobby both major parties to commit $25m funding to the Alliance (Watson^64^)
New issues raised by members of the public involved	80 biobanks in Western Australia; Avon Longitudinal Study of Parents and Children (ALPSAC); BC Biobank; BC Biolibrary; CARTaGENE; Generation Scotland; International HapMap Project; Kilifi Genetic Birth Cohort; Mayo Clinic Biobank; Personalized Medicine Research Project (PMRP); The Peninsula Research Bank; UK Biobank	Coors et al,^32^ Godard et al,^47^ Kimball et al,^16^ Lemke et al,^33^ Levitt,^56^ Levitt and Weldon,^70^ Marsh et al,^63^ McCarty et al,^12^ Molster et al,^35^ O'Doherty et al,^56^ Rotimi et al,^62^ Secko, 2008, Terry et al,^37^ Walmsley,^51^ O'Doherty and Burgess,^59^ Jenner et al,^53^ Haddow et al,^38^ Lemke et al^61^	‘The participants in our project wanted fair and equitable access, and wanted a voice in the process. 4 main areas of concern after first dialogue session: (1) access to HapMap data and therapies that result from it; (2) regulation of HapMap and biomedical research; (3) potential misuse of HapMap data; and (4) issues particular to race’ (Terry et al^37^)
Research design			
New research questions/outcomes	AFM; BCCTB; ICCon; International HapMap Project; PXE	Forrest et al,^34^ Terry et al,^37^ Terry et al,^43^ Wilcox et al,^21^ Rabeharisoa^42^	‘CAC members developed research questions’ (Terry et al^37^)
Study design & methods			
Proposal of strategies/methods	BCCTB; BioVU; International HapMap Project; Kilifi Genetic Birth Cohort; The Peninsula Research Bank; UK Biobank; Wales Cancer Bank	Levitt and Weldon,^69^ Marsh et al,^62^ Mitchell et al,^3^ Rotimi et al,^61^ Terry et al,^37^ Wilcox et al,^21^ Jenner et al,^52^ Lemke et al,^60^ Rabeharisoa^42^; AFM	‘Electronic consenting and volunteer consenting were both (individually) submitted to the [Lay Liaison and Ethics] group and only with their support have both schemes been progressed further for more detailed investigation and potential implementation.’ (Mitchell et al^3^)
Practicalities of participation	AFM; BCCTB; BioVU; International HapMap Project; Multisite keloid study; The Peninsula Research Bank; UK Biobank	Levitt and Weldon,^70^ Olaitan et al,^66^ Terry et al,^37^ Wilcox et al,^21^ Jenner et al,^52^ Rabeharisoa^42^	‘raise practical questions about study design, such as during a long study visit when can participants drink or visit the toilet? Taking time to consider these practicalities ensures that study visits run more smoothly’ (Jenner et al^53^)
Establishment of biobanks	AFM; PATH; PXE	Terry et al,^43^ Rabeharisoa,^42^ Mitchell et al^3^	‘Breast cancer survivors established the PATH Biobank in 2002 as a non‐profit organisation to collect human tumour and blood samples, together with patient data (and follow‐up data) at high ethical standards and under uniform SOPs. PATH aims to involve the breast cancer patients as much as possible in its work’ (Mitchell et al^3^)
On people			
People involved			
Trust	AFM; Multisite keloid study; PXE; Telethon Network of Genetic Biobanks; Wales Cancer Bank	Baldo et al,^41^ Olaitan et al,^67^ Terry et al,^43^ Rabeharisoa,^42^ NIHR Cancer Research Network (NCRN)^72^	‘A novel and essential component was the establishment of a community of trust — a gathering of individuals bound by the effects of mutations in the ABCC6 gene that underlies PXE, who could share their experiences’ (Terry et al^43^)
Education	AFM; Tasmania Biobank; Telethon Network of Genetic Biobanks	Baldo et al,^41^ Chalmers et al,^4^ Rabeharisoa^42^	‘Deliberative democracy brings to medical research and health care policy a method by which the community can gain standing in the development of policy on a range of issues previously dominated by researchers, lawyers and ethicists. The advantage of this method over previous ones is that it is a two‐way, iterative process of information exchange’ leading to ‘significant shifts in participants' thinking associated with access to information and dialogue with researchers’ (Chalmers et al^4^)
Skills	AFM; Nottingham Health Science Biobank; PXE	Mitchell et al,^3^ Terry et al,^43^ Rabeharisoa^42^	ongoing professional development for five PPI Advocates: ‘ne‐to‐one training and taken through the full life cycle of biobanking’ (Mitchell et al^3^)
Personal	AFM; Avon Longitudinal Study of Parents and Children (ALPSAC); BC Biobank; Generation Scotland; UK Biobank	Levitt,^56^ Terry et al,^43^ Walmsley,^22^ Haddow et al,^38^ Rabeharisoa^42^	‘focus group participants expressed frustrations at the moderators as [they] were asking about views on the implications of research yet to happen, which they had previously thought little about’ (Haddow et al^38^)
Further participation and involvement	AFM; BC Biolibrary; BCCTB; Nottingham Health Science Biobank; PATH; PXE; Wales Cancer Bank	Mitchell et al,^3^ O'Doherty and Hawkins,^24^ Terry et al,^43^ Wilcox et al,^21^ Lemke et al,^61^ Rabeharisoa^42^	‘A steering group [38 steering group members] was convened when the biobank was founded to ensure all relevant stakeholders were able to input into the formation and initiation of the WCB [...] Three lay members were patients, and one was a caregiver for a cancer patient. These four lay members went on to form the core of the patient and ethics committee’ (Mitchell et al^3^)
Researchers & biobank staff			
Understanding & conformity to guidelines	BC Biolibrary	Jenner et al,^53^ O'Doherty and Hawkins^24^	‘bringing lay representatives into the research arena helps to raise awareness of issues outside the academic culture box: lay members are more focussed on the practical aspects and outcomes of research and how it can affect patients and carers’ [‘PPI groups are better placed to decide whether what is being asked of them in research participation is reasonable and worthwhile. They will often raise issues that neither the originating investigators nor ethics committees had considered’] (Jenner et al^53^)
Research participants			
Clinical referrals	Nottingham Health Science Biobank; PATH	Mitchell et al^3^	‘PATH consults patients free of charge concerning the handling of their tumour tissue [...] They have right to decide what happens to their tumour. For these reasons, breast cancer patients may call the PATH office and receive support free of charge. The PATH staff consists of a physician and a biologist, thus allowing for competent consultancy’ (Mitchell et al^3^)
Facilitated relationships with researchers	AFM; CARTaGENE; Melbourne Genomics Health Alliance; Multisite keloid study; NuGene; The Peninsula Research Bank; Wales Cancer Bank	Lemke et al,^33^ Olaitan et al,^67^ O'Doherty and Burgess,^59^ Jenner et al,^53^ Lemke et al,^61^ Lemke et al,^33^ Watson,^64^ Rabeharisoa,^42^ NIHR Cancer Research Network (NCRN)^72^	‘shared community insights important in facilitating relationships and policy discussions between biobank researchers and research participant’ (Lemke et al^33^)
Shift in thinking	Multisite keloid study; Tasmania Biobank; Wales Cancer Bank	Chalmers et al,^4^ Olaitan et al,^67^ Rabeharisoa,^42^ NIHR Cancer Research Network (NCRN)^71^	‘Interaction with members of the oldest generation or children of old family members may be difficult when the culture of scientists and recruiters is different from that of the participants. To address these and other issues, our study involved research staff from the local community who were born and raised in the Yoruba culture.’ (Olaitan et al^67^)
Wider community			
Health promotion	AFM; Multisite keloid study; Nottingham Health Science Biobank; PATH; Wales Cancer Bank	Mitchell et al,^3^ Olaitan et al,^67^ Olaitan et al,^67^ Rabeharisoa^42^	‘Patients and their families also collaborate with specialists in the production of knowledge to further their understanding of their diseases and to explore therapeutic possibilities and different ways of caring for patients’ (Rabeharisoa^42^)
Education	CARTaGENE; Generation Scotland; International HapMap Project; Kilifi Genetic Birth Cohort; Melbourne Genomics Health Alliance; Multisite keloid study; Nottingham Health Science Biobank; NuGene; Telethon Network of Genetic Biobanks; Wales Cancer Bank	Filocamo et al,^40^ Marsh et al,^63^ Mitchell et al,^3^ Olaitan et al,^67^ Terry et al,^37^ O'Doherty and Burgess,^59^ Haddow et al,^38^ Lemke et al,^61^ Watson^63^	‘the PEGV Project made a strong effort to provide education in addition to policy development.’ (Terry et al^37^)
Wider engagement with science and research	International HapMap Project; Kilifi Genetic Birth Cohort; Melbourne Genomics Health Alliance; Mayo Clinic Biobank; Multisite keloid study; PATH; Telethon Network of Genetic Biobanks	Filocamo et al,^40^ Lemke et al,^33^ Marsh et al,^63^ Olaitan et al,^66^ Rotimi et al,^62^ Terry et al,^37^ Haddow et al,^38^ Lemke et al,^61^ Watson^64^	‘raising awareness, trust and interest of the general public in Biobanks’ (Filocamo et al^40^)
Impact on the wider research community			
Further public involvement in biobank			
Creation of Advisory Board	Alaska Area Specimen Bank (AASB); Mayo Clinic Biobank; Wales Cancer Bank	Mitchell et al,^3^ Parkinson et al,^39^ Lemke et al^61^	key outcome of the deliberation exercise was the deliberant's recommendation that Mayo Clinic establish the Biobank Community Advisory Board (Lemke et al^61^)
Creation of more opportunities for ongoing discussion with wider representation	Alaska Area Specimen Bank (AASB); Generation Scotland; Mayo Clinic Biobank; PXE; UC Biobank; Wales Cancer Bank	Dry et al,^69^ Mitchell et al,^3^ Olson et al,^36^ Parkinson et al,^39^ Terry et al,^43^ Haddow et al,^38^ Lemke et al^61^	working group recommended the establishment of ‘a forum for ongoing discussion and resolution of concerns and issues related to use of banked specimens’ (Parkinson et al^39^)
New agreements with patient organizations	AFM; PXE; Telethon Network of Genetic Biobanks	Baldo et al,^41^ Filocamo et al,^40^ Terry et al,^43^ Rabeharisoa^42^	‘AFM was also behind the recent creation of the Alliance Francaise des Maladies Rares (French Alliance for Rare Diseases), an umbrella organisation currently grouping together 80 patient organisations’ (Rabeharisoa^42^)
Participation in recruitment of new biobank staff	Avon Longitudinal Study of Parents and Children (ALPSAC)	Levitt^56^	‘one participant reported enthusiastically on her role which included being involved in the interviewing process for new staff’ (Levitt^56^)
Lasting and trusting research partnerships	80 biobanks in Western Australia; AFM; Alaska Area Specimen Bank (AASB); Avon Longitudinal Study of Parents and Children (ALPSAC); BCCTB; Kaiser Permanente; Kilifi Genetic Birth Cohort; Multisite keloid study; PXE; Tasmania Biobank; Telethon Network of Genetic Biobanks; UK Biobank; Wales Cancer Bank	Baldo et al,^41^ Boyer et al,^60^ Chalmers et al,^4^ Filocamo et al,^40^ Levitt,^56^ Levitt and Weldon,^70^ Marsh et al,^63^ Molster et al,^35^ Olaitan et al,^67^ Parkinson et al,^39^ Terry et al,^43^ Wilcox et al,^21^ Lemke et al,^61^ Rabeharisoa,^42^ NIHR Cancer Research Network (NCRN)^72^	‘The practical suggestions made in terms of increasing trust consisted, on the one hand, of safeguards and limitations to the power of any one interest group and, on the other, to being able to trust those in charge as “safe hands”.’ (Levitt and Weldon^69^)
Transparency and accountability	AFM; BCCTB; Multisite keloid study; Tasmania Biobank; Telethon Network of Genetic Biobanks; UK Biobank; Wales Cancer Bank	Chalmers et al,^4^ Filocamo et al,^40^ Levitt and Weldon,^70^ Molster et al,^35^ Olaitan et al,^67^ Wilcox et al,^21^ Lemke et al,^61^ Rabeharisoa,^42^ NIHR Cancer Research Network (NCRN)^72^	‘Visits to kings, chiefs, churches and mosques were useful to convince the community that blood and saliva samples would not be used for voodoo or juju. It was important and reassuring to be accompanied by research participants from their community at such meetings to demonstrate that nothing bad had happened to them since recruitment’ (Olaitan et al^67^)
Public representation	80 biobanks in Western Australia; AFM; BC Biolibrary; BCCTB; CARTaGENE; Tasmania Biobank; The Peninsula Research Bank; Wales Cancer Bank	Chalmers et al,^4^ Godard et al,^46^ Mitchell et al^3^; Molster et al,^35^ O'Doherty et al,^57^ Wilcox et al,^21^ Jenner et al,^53^ Lemke et al,^61^ Rabeharisoa^42^	‘Deliberative democracy brings to medical research and health care policy a method by which the community can gain standing in the development of policy on a range of issues previously dominated by researchers, lawyers and ethicists. […] Another advantage of this deliberative democracy methodology is that it permits effective consultation with cohorts of people who are traditionally difficult to engage, including people who live in remote locations, who are from lower socio‐economic strata and who have lower literacy levels’ (Chalmers et al^4^)
Contributions to knowledge			
Mentoring other biobanks/advocacy groups	AFM; PXE	Terry et al,^43^ Rabeharisoa^42^	mentoring other advocacy groups (creation of the Interactive Guide to Building Advocacy Organizations) (Terry et al^43^)
Media presence, manuscripts and conference presentations	AFM; International HapMap Project; Melbourne Genomics Health Alliance	Terry et al,^37^ Terry et al,^43^ Lemke et al,^61^ Watson,^64^ Rabeharisoa^42^	‘providing access to actual patients and their stories through CAG patient networks ensured media uptake of press releases, driving public interest in the Alliance’ (Watson^63^)
Dissemination and further adoption of involvement methods in STPs	AFM; PXE	Terry et al,^43^ Rabeharisoa^42^	‘creation of the Genetic Alliance Biobank (a coalition of over 600 disease advocacy organizations, using its infrastructure as a repository for their model and methods)’ (Terry et al^43^)
